# 云南省昆明市城市癌症早诊早治项目：肺癌高危人群评估及筛查效果分析

**DOI:** 10.3779/j.issn.1009-3419.2020.101.30

**Published:** 2020-07-20

**Authors:** 艳苹 林, 洁 马, 萌 吴, 海 周, 彦霓 陆, 泳村 岑, 中琴 袁, 泽超 梅, 云超 黄, 永春 周

**Affiliations:** 650118 昆明，云南省肿瘤医院/昆明医科大学第三附属医院/云南省癌症中心 Department of Yunnan Cancer Center, Yunnan Cancer Center/Yunnan Cancer Hospital/The Third Affiliated Hospital of Kunming Medical University, Kunming 650118, China

**Keywords:** 肺肿瘤, 筛查, 效果, 云南, Lung neoplasms, Screening, Effect, Yunnan

## Abstract

**背景与目的:**

肺癌是最常见的恶性肿瘤，预后较差，5年生存率较低，早期筛查是肺癌防治的重要措施。目前，不同国家和地区发布了相应的肺癌筛查指南，但我国仍缺乏基于中国人群研究的指南。因此，国家癌症中心在全国启动了多中心的城市癌症早诊早治项目研究。本研究分析了城市癌症早诊早治项目中云南省单中心的肺癌高危人群评估模型及临床筛查应用效果，为探索适合我国国情的肺癌高危人群评估模型及制定和更新中国人群肺癌筛查指南提供一定的参考依据。

**方法:**

采取整群抽样的方法，于2015年1月-2019年12月对云南省昆明市4个主城区36个街道办事处165, 337人进行问卷调查及肺癌风险评估，评估为高风险者进行胸部低剂量计算机断层扫描（low-dose computed tomography, LDCT）筛查。同时对所有参与者进行主动随访和被动随访，获得详细的临床结局，进行统计分析。

**结果:**

整体人群5年间经病理确诊肺癌患者264例，总体肺癌发生率为0.16%（264/165, 337），高风险组（0.31%, 116/37, 914）高于非高风险组（0.12%, 148/127, 423），差异有统计学意义（*P* < 0.001）。不同性别和不同年龄的亚组分析显示高风险组肺癌发生率均高于非高风险组，均有统计学差异（*P* < 0.001），但在未进行LDCT筛查组无统计学差异（*P*=0.73）。肺癌高危人群评估模型的敏感性为43.94%（116/264），特异性为77.10%（127, 275/165, 073）。筛查组早诊率为72.97%（54/74），明显高于非筛查组的28.48%（43/151）。

**结论:**

国家重大公共卫生服务项目城市癌症筛查项目肺癌高危人群评估模型能有效检出高风险人群，提高肺癌早诊率。

## Abstract (en)

**Backgroud and objective:**

Lung cancer is the most common neoplasmas with a poor prognosis and a low 5-year survival rate. Early screening is an important measure for the prevention and treatment of lung cancer. At present, different countries have issued corresponding lung cancer screening guidelines, but China still lacks guidelines based on Chinese population research. Therefore, the National Cancer Center launched a Multi-center Cancer Screening Program in Urban China. This study analyzed the evaluation of lung cancer risk assessment model and screening effect in urban China of Yunnan, so as to explore the evaluation model of high-risk lung cancer population suitable for China's national conditions and develop lung cancer screening guidelines for Chinese.

**Methods:**

A questionnaire survey and lung cancer risk assessment were conducted on 165, 337 people in 36 street offices in 4 main urban areas of Kunming, Yunnan Province, using cluster sampling method from January 2015 to December 2019. People with high-risk of lung cancer conducted low-dose computed tomography (LDCT) screening of chest. What's more, all participants were followed up by active or passive follow-up.

**Results:**

There were 264 patients were diagnosed lung cancer by pathology, and the overall incidence of lung cancer was 0.16% (264/165, 337). The high-risk group (0.31%, 116/37, 914) was higher than the non-high-risk group (0.12%, 148/127, 423), and the difference was statistically significant (*P* < 0.001). The incidence of lung cancer in the high-risk group was higher than the non-high-risk group among the male, female, and lower 50-year-old or more than 50-year-old subgroups, with statistical differences (*P* < 0.001), but there was no statistical difference in the group without LDCT screening (*P*=0.73). The sensitivity of the lung cancer high-risk population assessment model was 43.94% (116/264) and the specificity was 77.10% (127, 275/165, 073). The early diagnosis rate of the screening group was 72.97% (54/74), which was significantly higher than that of the non-screening group [28.48% (43/151)].

**Conclusion:**

The lung cancer high-risk population assessment model of National Key Public Health Program: Cancer Screening Program in Urban China can detect high-risk populations and improve the early diagnosis rate of lung cancer effectively.

肺癌是最常见的恶性肿瘤，其在全球及中国的发病率和死亡率均居恶性肿瘤首位^[1, 2]^。2019年我国恶性肿瘤登记数据^[2]^显示，新发肺癌78.7万例，发病率57.26/10万；肺癌死亡63.1万例，死亡率45.87/10万。2015年，中国癌症生存分析数据显示肺癌的发病和死亡例数分别达733, 300例和610, 200例，发病率和死亡率接近，其主要原因是晚期病例占比较大，无法手术治疗，预后极差，5年生存率仅为16.1%^[3]^。

2011年，美国国家肺癌筛查随机对照试验（National Lung Screening Trial, NLST）结果显示，高危人群进行低剂量计算机断层扫描（low-dose computed tomography, LDCT）较胸部X线片筛查可使肺癌死亡率降低20%^[4]^。可见，筛查是提高肺癌生存率、降低死亡率的重要措施。此外，其他多个国家和地区也进行了肺癌筛查相关的实验研究，如荷兰-比利时随机肺癌筛查（NELSON试验）^[5]^英国肺癌筛查^[6]^（UK Lung Cancer Screening, UKLS）以及匹兹堡肺部筛查^[7]^等研究，均在肺癌高危人群评估模型、筛查方案等方面进行了研究。但是目前，我国尚缺乏大样本的肺癌筛查随机对照研究，由于不同国家和地区肺癌的流行情况、医疗保健系统及人群对肺癌筛查的接受程度或依从性并不相同，其他国家的研究结果能否在中国推广尚无证据支持。因此，2009年国家医改重大专项“农村癌症早诊早治”项目将肺癌纳入试点，启动了我国肺癌高危人群筛查工作^[8]^。国家重大公共卫生服务项目“城市癌症早诊早治项目”启动了大规模的城市肺癌筛查工作。城市癌症早诊早治项目自2012年启动至今，先后纳入全国26个省份、51个城市，针对全国城市范围内高发的五大类癌症，即肺癌、乳腺癌、结直肠癌、上消化道癌（食管癌和胃癌）以及肝癌开展高危人群评估、临床筛查、随访和早诊早治等工作，取得了显著成效^[9]^。项目启动以来，全国各项目参加省份和城市总结报道了很多筛查数据结果，但仍缺乏后续随访确诊恶性肿瘤情况及对高危人群评估模型和临床筛查方法效果的客观评价。本研究以云南省2015年1月-2019年12月5年间项目开展的真实世界数据为基础，进一步分析和评估肺癌高风险模型及临床筛查效果，为项目最终筛查效益的分析及我国肺癌筛查指南的制定和更新提供参考依据。

## 1 材料与方法

### 1.1 研究对象

2015年1月-2019年12月先后纳入昆明市4个主城区（西山区、官渡区、五华区、呈贡区）的常住居民，纳入标准：①本市户籍常驻人口或本地居住3年及以上；②年龄在40岁-74岁之间；③签署项目知情同意书。排除标准：①无法完成知情同意书；②需要氧供应才能保证呼吸功能；③仰卧时不能把胳膊放在头上；④胸部或背部有金属植入物或金属设备（心脏起搏器等），影响肺部成像；⑤有癌症史；⑥既往肺部手术史，不包括经皮肺穿刺。

### 1.2 研究方法

#### 1.2.1 危险因素问卷调查

所有调查对象在各街道社区专人指导下自行填写危险因素调查问卷或由经过专业培训的调查员询问调查对象后填写问卷。危险因素调查问卷包括基本信息、饮食习惯、生活环境、生活方式和习惯、心理和情绪、疾病既往史、恶性肿瘤家族史、女性生理和生育史等。

#### 1.2.2 高危人群评估

工作人员质控调查问卷后录入国家癌症中心开发的高危人群评估系统初筛肺癌高危人群。该系统以“哈佛癌症风险指数”理论^[10]^为基础，依据近20年来我国常见癌症流行病学资料，通过多学科专家小组讨论达成共识的方法，确定我国成年人癌症发病的主要危险因素及相关赋值，应用哈佛癌症风险指数工作小组推荐的公式，研发出的适合我国人群的个体癌症风险综合评价体系^[11]^。城市癌症早诊早治项目高危风险评估系统引入肺癌相关因素为吸烟指数、日常平均新鲜蔬菜摄入量、长期生活环境空气污染、日常体育锻炼情况、慢性呼吸系统疾病史、肺癌家族史、被动吸烟史等。评估为高风险的直入条件为吸烟指数≥400（每日吸烟的支数×吸烟的年数），且年龄≥50岁；超过20年接受二手烟（被动吸烟）者；有较长期职业暴露史者（石棉、铍、铀、氡等）。

#### 1.2.3 临床筛查

问卷调查后评估为肺癌高风险人群至云南省肿瘤医院进行胸部LDCT扫描，扫描层厚5 mm，层间距5 mm，重建层厚1.0 mm-1.25 mm连续（层间隔为0），扫描范围从肺尖到肋膈角（包括全部肺），受检者吸气末一次屏气完成扫描。由高年资（3年以上）放射科医师出具检查报告并填写筛查记录表。

#### 1.2.4 随访

本研究对所有参加危险因素问卷评估对象进行被动随访（利用肿瘤登记数据库和医院病案管理信息系统平台，通过唯一识别的身份证号码进行匹配），同时对筛查阳性人群进行电话主动随访，详细记录肺癌确诊患者的人口学资料及诊疗信息。

#### 1.2.5 相关指标定义

① 高风险率=评估为肺癌高风险例数/问卷调查数×100%；②早期肺癌：Ⅰ期肺癌病例；③早诊率=早期肺癌病例数/确诊肺癌病例数。

### 1.3 统计学分析

所有数据应用SPSS 19.0统计软件建立数据库及统计分析，计数资料采用率或构成比表示，采用卡方检验比较高风险组和非高风险组肺癌检出率的差异以及不同性别、年龄和是否进行LDCT筛查亚组之间肺癌检出率的差异，双侧检验，检验水准α=0.05。

## 2 结果

### 2.1 基本情况

2015年1月-2019年12月，云南省进行城市癌症早诊早治项目防癌危险因素问卷调查165, 337人，其中男性75, 755人，占45.82%；女性89, 582人，占54.18%；男女比例0.85:1；40岁-50岁67, 127人，占40.60%；51岁-60岁52, 958人，占32.03%；61岁-70岁41, 485人，占25.09%；≥71岁3, 767人，占2.28%。评估出肺癌高危人群37, 914人，高风险率为22.93%。通过临床筛查、主动随访及被动随访，5年间经病理确诊肺癌患者264例，总体肺癌检出率为0.16%（264/165, 337），其中肺癌高风险人群组确诊肺癌116例（参加临床筛查人群确诊88例，未参加临床筛查确诊28例），非高风险人群确诊肺癌148例。详见图 1。

**1:**
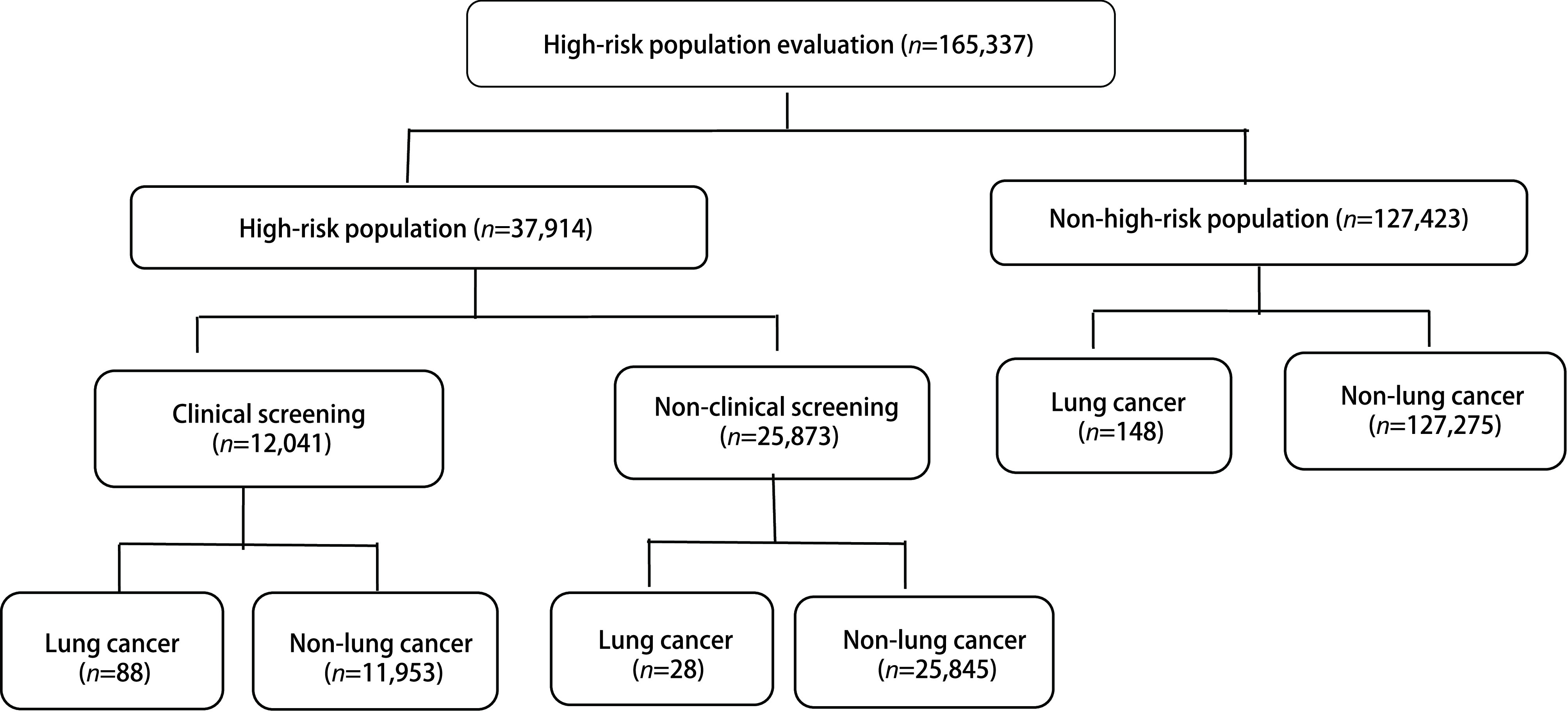
肺癌筛查研究流程图 The flow chart of the lung cancer screening

### 2.2 高风险组和非高风险组肺癌患病情况比较

5年间经病理确诊肺癌患者264例，总体肺癌发生率为0.16%（264/165, 337），高风险组肺癌发生率（0.31%, 116/37, 914）高于非高风险组（0.12%, 148/127, 423），差异有统计学意义（*χ*^2^=212.24, *P* < 0.001）。此外，对不同性别、年龄分组及是否进行LDCT筛查进行亚组分析提示，在男性（*χ*^2^=14.39, *P* < 0.001）、女性（*χ*^2^=58.62, *P* < 0.001）、 < 50岁（*χ*^2^=46.62, *P* < 0.001）及≥50岁（*χ*^2^=153.08, *P* < 0.001）亚组间高风险组肺癌发生率均高于非高风险组，有统计学差异；但在未进行LDCT筛查组中高风险组与非高风险组的肺癌发生率无统计学差异（*χ*^2^=0.12, *P* =0.73）。详见表 1。

**1:** 高风险组与非高风险组肺癌发生率的亚组比较分析 Subgroup analysis of lung cancer incidence between high-risk and non-high-risk groups

Characteristics	The high-risk group (*n*=37, 914)				The non-high-risk group (*n*=127, 423)			*χ*^2^	*P*
	*n*	Lung cancer [*n* (%)]	Non-lung cancer [*n* (%)]		*n*	Lung cancer [*n* (%)]	Non-lung cancer [*n* (%)]		
Gender									
Male	23, 741	67 (0.28)	23, 674 (99.72)		52, 014	79 (0.15)	51, 935 (99.85)	14.39	< 0.001
Female	14, 173	49 (0.35)	14, 124 (99.65)		75, 409	69 (0.09)	75, 340 (99.91)	58.62	< 0.001
Age (yr)									
< 50	13, 693	26 (0.19)	13, 667 (99.81)		47, 662	12 (0.03)	47, 650 (99.97)	46.62	< 0.001
≥50	24, 221	90 (0.37)	24, 131 (99.63)		7, 976	136 (1.71)	7, 840 (98.29)	153.08	< 0.001
LDCT screening									
Yes	12, 041	88 (0.73)	11, 953 (99.27)		NA	NA			
No	25, 873	28 (0.11)	25, 845 (99.89)		127, 423	148 (0.12)	127, 275 (99.88)	0.12	0.730
LDCT: low-dose computed tomography; NA: not available.									

### 2.3 肺癌高危人群评估模型的敏感性和特异性分析

共37, 914例评估为肺癌高风险人群，其中116例确诊肺癌；共127, 423例评估为肺癌非高风险，其中148例确诊肺癌。肺癌高危人群评估模型的敏感性为43.94%（116/264），特异性为77.10%（127, 275/165, 073），详见表 2。

**2:** 肺癌高危人群评估模型的敏感性和特异性 The sensitivity and specificity of the high-risk assessment model of lung cancer

The high-risk assessment	Lung cancer		Sensitivity	Specificity
	Yes	No		
High-risk of lung cancer	116	37, 798	43.94%	77.10%
Non-high-risk of lung cancer	148	127, 275		

### 2.4 高风险组和非高风险组肺癌确诊分期比较

本组随访确诊的264例肺癌患者中，追踪到确切的肺癌病理分期患者225例，其中Ⅰ期97例，Ⅱ期29例，Ⅲ期44例，Ⅳ期55例。筛查组以早期患者为主，非筛查组以晚期患者为主。筛查组Ⅰ期/Ⅱ期占比明显高于非筛查组，而Ⅲ期/Ⅳ期患者占比明显低于非筛查组。详见图 2。筛查组早诊率为72.97%（54/74），明显高于非筛查组的28.48%（43/151）。

**2:**
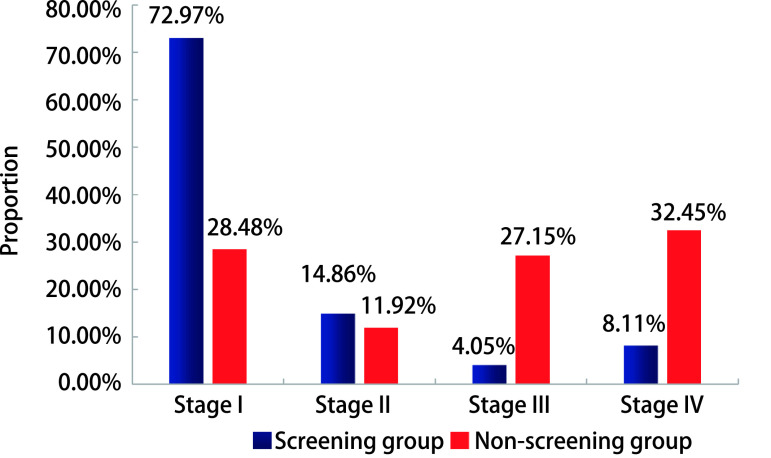
筛查组和非筛查组肺癌患者分期 The stage of lung cancer in screening and non-screening groups

## 3 讨论

城市癌症早诊早治项目采用防癌风险因素问卷调查，通过癌症风险相关系数评估出肺癌高风险人群，并对高风险人群进行LDCT临床筛查。本研究高风险组（0.31%）和非高风险组（0.12%）肺癌发生率略低于徐州市0.42%和0.17%的研究结果^[12]^，但差异较小。本研究总体分析及不同性别和不同年龄亚组分析提示高风险组肺癌的发生率高于非高风险组，差异有统计学意义，体现了危险因素评估高风险人群是有效的，这与徐州市单中心的研究结果^[12]^一致。但在高风险人群中存在部分人群未进行临床筛查，高风险组肺癌发生率高于非高风险组的结果是否由于参加临床筛查导致？徐州市的研究结果^[12]^显示，肺癌高风险未筛查人群和非高风险未筛查人群的肺癌发生情况存在统计学差异，证实了高危风险评估在肺癌的评估工作中是有效的。但本研究也对非筛查人群中高风险组与非高风险组的肺癌发生率进行比较，遗憾的是两组间无统计学差异。这可能与各地区危险因素问卷调查质控情况、筛查技术水平及筛查顺应性等有关。目前全国各省单中心的研究报道有限，全国多中心的研究结果也未发布。由此可见，高危风险评估模型对肺癌高危人群的评估效果需要全国更多分中心研究和多中心研究综合结果的统计数据进一步证实。

本项目采用的肺癌高危人群评估模型灵敏度为43.94%，特异度为77.10%，灵敏度高于徐州市^[12]^的35.29%，特异度低于略低于徐州市的81.70%。这可能与不同地区低剂量筛查CT分辨率、影像诊断医师诊断水平以及高危问卷评估工作方式等差异有关。但整体来看，高危人群评估模型的敏感性不理想，这可能是因为危险因素问卷调查内容多为主观问题，调查对象在填写时存在一定偏倚，同时质量控制也是重要影响因素，需加强质量控制及完善评估模型改进，从而提升其评估效率。

多数LDCT筛查的研究根据年龄和吸烟量来选择高危人群，可能导致女性、不吸烟人群以及其他危险因素人群的评估存在偏倚；越来越多的证据^[13, 14]^表明，完善的危险预测模型可能有助于更为精准地筛选适合肺癌LDCT筛查的高危个体。美国一项研究^[15]^在利用PLCO试验对照的数据建立一种肺癌危险预测模型，并在PLCO筛查组和NLST参与者及美国50岁-80岁的吸烟者中进行了验证，结果发现，与美国预防服务工作组标准相比，基于模型选择高危人群进行筛查可避免更多的肺癌死亡，并可降低避免肺癌死亡所需的筛查人数。分子标志物有助于鉴别肺癌高危人群，其与LDCT筛查的联合应用可能降低过高的假阳性结果^[16]^。研究^[17]^显示，鳞状细胞癌相关抗原（squamous cell carcinoma antigen, SCC）+神经元特异性烯醇化酶（neuron‐specific enolase, NSE）+癌胚抗原（carcinoembryonic antigen, CEA）+细胞角蛋白19片段（cytokeratin 19 fragment, CYFRA21-1）联合筛查肺癌的检出率和早检率较高，成本尚可接受，通过选择高危人群，可以进一步提高筛查效率降低筛查成本。NLST试验已经建立了包括血、痰和尿液样本的生物样品库，以便发现和验证确定肺癌高危个体、区分结节良恶性以及预测肿瘤生物学行为的标志物^[18]^。是否可在现有评估模型基础上增加标志物等客观指标或改进现有评估内容，进一步提高评估模型敏感性和特异性，需要进一步研究。

此外，课题组前期进行了2005年-2014年以来云南省肺癌临床流行病学变化特征分析^[19]^显示，近10年来云南地区肺癌流行病学变化特征主要为女性、不吸烟患者比例增加，临床分期较晚、Ⅲ期/Ⅳ期患者比例逐年增加，晚期患者占比较大仍然是本地区肺癌防治工作的重点和难点。云南省2012年-2015年肺癌患者预后生存分析研究^[20]^显示是否进行肺癌体检筛查、临床分期是肺癌患者的重要预后影响因素，筛查人群的1年、2年、3年生存率分别为80%、70%、60%，而非筛查人群仅为50%、30%、15%。可见体检筛查是提高早期患者比例、改善肺癌患者预后生存的重要手段。虽然本研究提示评估手段敏感性和特异性并非很高，但结果显示筛查组早诊率明显高于非筛查组，筛查组Ⅰ期/Ⅱ期患者占比较大，大多数患者进行手术治疗后无需进一步行放化疗，生活质量得到明显提升，可见临床筛查是提高肺癌早诊率的重要手段。目前国家癌症中心城市癌症早诊早治项目组也对既往工作进行数据分析、经验总结，并对高危人群危险因素调查问卷进行改进，期待进一步长期持续跟踪随访，后续能对比研究筛查项目实施后肺癌患者的临床分期及生存情况的改善。期待全国多中心的研究结果为我国肺癌筛查指南的制定和更新提供更多循证医学依据。
